# IL1B Induced Smad 7 Negatively Regulates Gastrin Expression

**DOI:** 10.1371/journal.pone.0014775

**Published:** 2011-03-22

**Authors:** Dipanjana Datta De, Sumana Bhattacharjya, Meenakshi Maitra, Arindam Datta, Abhijit Choudhury, G. K. Dhali, Susanta Roychoudhury

**Affiliations:** 1 Molecular and Human Genetics Division, Indian Institute of Chemical Biology, Council of Scientific and Industrial Research, Kolkata, India; 2 Department of Pediatrics, University of Texas Southwestern Medical Center, Dallas, Texas, United States of America; 3 Departments of Medicine and Gastroenterology, Institute of Postgraduate Medicine and Experimental Research, Kolkata, India; Sun Yat-Sen University, China

## Abstract

**Background:**

*Helicobacter pylori* elicited IL1B is one of the various modulators responsible for perturbation of acid secretion in gut. We have earlier reported that IL1B activated NFkB downregulates gastrin, a major modulator of acid secretion. However, we hypothesized that regulation of gastrin by IL1B would depend on the cell's ability to integrate inputs from multiple signaling pathways to generate appropriate biological response.

**Principal Finding:**

In this study, we report that IL1B induces Smad 7 expression by about 4.5 fold in gastric carcinoma cell line, AGS. Smad 7 resulted in transcriptional repression of gastrin promoter by about 6.5 fold when co -transfected with Smad 7 expression vector and gastrin-promoter luciferase in AGS cells. IL1B inhibited phosphorylation of Smad 3 and subsequently interfered with nuclear translocation of the positive Smad complex, thus occluding it off the gastrin promoter. *IL1B* promoter polymorphisms *(-511T/-31C IL1B*) are known to be associated with *H. pylori* associated gastro-duodenal ulcer. We observed that IL1B expressed from *-31T* promoter driven IL1B cDNA elicited 3.5 fold more Smad 7 than that expressed from the *IL1B-31C* variant in AGS cells. This differential activation of Smad 7 by IL1B promoter variants translated into differential downregulation of gastrin expression. We further analyzed Smad 7, NFkB, IL1B and gastrin expression in antral gut biopsy samples of patients with *H. pylori* associated duodenal ulcer and normal individuals. We observed that individuals with duodenal ulcer had significantly lower levels of IL1B, Smad 7, NFkB and corresponding higher level of gastrin expression.

**Conclusion:**

Pro-inflammatory cytokine IL1B repress gastrin expression by activating Smad 7 and subsequent inhibition of nuclear localization of Smad 3/4 complex. Polymorphic promoter variants of IL1B gene can modulate the IL1B expression which resulted in differential activation Smad 7 and consequent repression of gastrin expression, respectively. Analysis of *H. pylori* infected duodenal ulcer patient's gut biopsy samples also supported this observation.

## Introduction

Almost half of the world's population harbors *Helicobacter pylori* infection in the gastric mucosa [1]. A significant proportion of these infected individuals develop clinically relevant gastritis and some of these proceed to develop either gastric ulcers, gastric carcinoma or a low grade B- cell lymphoma [2], [3]. About 10–15% of individuals chronically infected with *H. pylori* develop antral predominant gastritis that predisposes them to develop duodenal ulcer [4]. Duodenal ulcer is characterized by increased basal and stimulated acid secretion which results due to perturbation of its major modulators, the gastrin-somatostatin hormone axis [5]. Pentagastrin-stimulated peak acid output, an indicator of functional parietal cell mass, is increased in *H. pylori* infected duodenal ulcer patients [6]. Gastrin Releasing Peptide-stimulated peak acid output, an indicator of the stomach's functional response to endogenous gastrin is also increased in these patients [6]. The mechanism of regulation of acid secretion on *H. pylori* infection remains unknown, but various reports suggest that cytokines might play a major role in this regulation [7]. The gastric mucosal inflammatory response to *H. pylori* involves increased synthesis of cytokines like IFN-γ, TNF-α, Interleukin -12 (IL12) and Interleukin 1Beta (IL1B) [8]. Genetic studies have suggested that polymorphisms in these genes are associated with *H. pylori* mediated gastro duodenal diseases [9], [10]. However, the molecular cue by which theses immunological messengers affect the downstream mediators of acid modulation is not well established. It has been reported that Interferon-γ suppresses somatostatin expression resulting in inhibition of IL4 production, which otherwise is stimulated by somatostatin to reverse *H. pylori*-induced gastritis [11]. The pro-inflammatory cytokine IL1B is known to negatively affect acid secretion in gut [12], [13], [14]. *In vivo* studies in human patients have established that the IL-1beta gene polymorphisms are related to hypochlorhydria and increased risk of gastric cancer in the presence of *H.pylori* infection [9].

We have previously reported that pro-inflammatory cytokine IL1B down-regulates gastrin via NFkB in a dose dependent manner [12]. This IL1B mediated gastrin inhibition was found to be reversed to about 40% by pharmacological or peptide inhibitors of NFkB [12]. Thus, inhibition of NFkB activation did not totally abrogate IL1B mediated gastrin repression. It is well established that such biological response of a gene by an upstream stimuli would depend upon integration of a number of pathways. The partial recovery of gastrin expression by NFkB inhibitors thus indicated the possibility of other independent IL1B mediated repression pathways [10]. In the present study, we have delineated an alternative orchestra of signaling events that would also lead to IL1B mediated attenuation of gastrin expression. We have also validated our observation in gut biopsy samples of *H. pylori* infected duodenal ulcer patients.

## Materials and Methods

### Subjects

The subjects included in this study were 20 unrelated individuals, [*H. pylori* infected individuals with duodenal ulcer (HP+U+ = 12), *H. pylori* infected asymptomatic individuals (HP+U− = 8)] who attended routine endoscopy at the SSKM hospital, Kolkata, India from August 2007 to April 2008. Gastric antral biopsy samples were collected from these individuals and further molecular and biochemical analysis was done. *H. pylori* status of the subjects was detected by rapid urease test, and confirmed by microscopy and PCR analysis for bacteria specific CAG pathogenicity region from the gastric biopsy samples (data not shown). The diagnosis of duodenal ulcer was established on the basis of conventional clinical and endoscopic findings. No subject had received treatment for *H. pylori* infection. Patients taking non-steroidal anti-inflammatory drugs or receiving antisecretory therapy, as well as those with gastric carcinoma were excluded from the study. Duodenal ulcer patients with gastrointestinal bleeding or suffering from osteoarthritis, cardiovascular diseases were also excluded. The biopsy samples were homogenized in Trizol (Invitrogen, Life Technologies, Carlsbad, USA) for RNA preparation. *Smad 7* and *IL1B* expression levels were analyzed in these samples by real time PCR. The biopsy tissue from same individuals were also homogenized in Lysis buffer (Tris, pH 8.0, EDTA, NaCl, NP40, Triton X, Protease inhibitor cocktail) and subjected to either ELISA or Western blot analysis for estimation of gastrin (G-17) (Gastrin ELISA KIT, Assay Design Inc., Ann Arbor, USA) and NFkB respectively. The protein was estimated by Bradford reagent (Sigma Aldrich, St. Louis, USA) according to manufacturer's protocol.

### Ethics statement

Prior to sample collection, written informed consent was obtained from each individual, which was approved by the Ethical Committee of the SSKM hospital, Kolkata.

### Cell Culture, transfection and treatment with IL1B, TGF beta, NBD, si-RNA

AGS cells, (3.2×10^6^) maintained in RPMI 1640 medium (Gibco BRL, Life Technologies, Grand Island, USA) were transiently transfected in duplicates with different amounts of DNA as required for the specific experiment by using Lipofectamine2000 Reagent (Invitrogen, Life Technologies, Carlsbad, USA) according to the manufacturer's protocol. PGL3 control vector (Promega, Madison, USA) was used as control for luciferase assay and beta-galactosidase plasmid vector (Promega, Madison, USA) was used as transfection control. AGS cells were treated with varying amounts (0–10 ng/ml) of recombinant IL1B (Sigma Aldrich, St. Louis, USA) for two and a half hours and harvested for RNA preparation, luciferase assay and Western blot analysis. AGS cells were also treated with 6 ng/ml TGF beta (eBioscience Inc, San Diego, USA) for 20 hours. AGS cells were incubated with 150 µM/ml of nemo binding inhibitory peptide (NBD) (Calbiochem-Novabiochem, San Diego, USA) after 24 hours of transfection and harvested for luciferase and Western blot analysis after 24 hours of NBD treatment. Various siRNA constructs directed against Smad 7 (sc-36508, Santa Cruz Biotechnology, CA and scrambled control (Ambion) were used at a final concentration of 80 nM.

### Quantitative Real Time PCR

RNA was prepared from recombinant IL1B treated AGS cells according to the manufacturer's protocol by Trizol method (Invitrogen, Life Technologies, Carlsbad, USA). cDNA was prepared by random hexamer (Invitrogen, Life Technologies, Carlsbad, USA) using MMLV RT (Promega, Madison, USA). *Smad 7* and *IL1B* mRNA expression was determined by real time RT PCR in the ABI 7500 Fast (Applied Bio-systems Inc, Life Technologies, Foster City, USA) using the SYBR green technology (Applied Biosystems Inc, Life Technologies, Foster City, USA). The primers used are as follows: *SMAD7* forward (F), 5′-GCCTCGGACAGCTCAATTCG-3′ and reverse (R), 5′-CGTCCACGGCTGCTGCATAA-3′; IL1B forward (F) 5′-AAACAGATGAAGTGCTC CTTCCAGG-3′ and reverse (R), 5′-TGGAGAACACCACTTGTTGCTCCA-3′, *Beta-actin* forward (F), 5′-GGATGCAGAAG GAGATCACTG-3′, and reverse (R), 5′-CGATCCA CACGGAGTACTTG-3′. Threshold cycle C_T_ of duplicate samples was determined using the ABI 7500 Fast software (Applied Biosystems Inc, Life Technologies, Foster City, USA). The level of *Smad 7* and *IL1B* was normalized to *Beta -actin* levels by calculating the Δ C_T_ value which is the C_T_ (threshold cycle) of the endogenous control (*Beta-actin*) subtracted from the C_T_ of the target gene (*Smad 7/IL1B)*. The fold difference in *Smad 7* and *IL1B* expression calculated using the formula 2 ^–ΔΔC^_T_.

### Western Blot analysis

Equal amount of total protein prepared from IL1B treated AGS cells (3.2×10^6^ cells) were subjected to Western blot analysis using antibodies against Smad 7, p-Smad 3, Smad 3 (Santa Cruz Biotechnology Inc, Santa Cruz, USA), or Beta -actin (Sigma Aldrich, St. Louis, USA). The immuno complex was detected by staining with HRP-conjugated secondary antibody (Sigma Aldrich, St. Louis, USA). The band intensities were quantified by using the image analysis software ImageJ (http://rsb.info.nih.gov/ij/index.html). The integrated density of each band was normalized to the corresponding human Beta-actin band.

### Immunofluorescence Assay

For immunefluorescence studies, AGS cells were cultured on coverslips placed in 35 mm plates. The cells were incubated with varying concentrations of recombinant IL1B protein for 30 minutes and then washed with phosphate buffered saline (PBS). The coverslips were overlaid with methanol fixative for 20 minutes at –20°C. Then cover slips were washed with PBS and cells were permeabilized with 0.5% Triton-X in PBS for 25 minutes at room temperature, and nonspecific binding sites were blocked with 1% bovine serum albumin in PBS. Finally the cells were washed with PBS and incubated with rabbit polyclonal antibody against Smad 3 (Santa Cruz Biotechnology Inc, Santa Cruz, USA), used at 1∶50 dilution, for overnight. Cells were washed with PBS to remove unbound antibody, and bound rabbit immunoglobulin G (IgG) was detected with goat anti-rabbit IgG-fluorscein isothicyanate (FITC) conjugate (Santa Cruz Biotechnology Inc, Santa Cruz, USA) used at 1∶400 dilution. Unbound conjugate antibody was removed by washing in PBS. Stained cells were mounted with sodium phosphate buffer containing 10% glycerol and DABCO (Sigma Aldrich, St. Louis, USA) as an antiquencher and examined at 40X in fluorescence microscopy LSM-510 (Carl Zeiss, Germany).

### Luciferase Assay

The cells were lysed in the luciferase cell culture lysis buffer provided with the Luciferase Assay Kit (Promega, Madison, USA) and 15 µl of supernatant was analyzed for firefly luciferase activity. Luminescence was measured as relative light units (RLU), taking the reading of luciferase assay substrate alone and then with lysate in GlowMax 20/20 luminometer (Promega, Madison, USA). The total protein concentration in each lysate was determined with a protein assay kit (Sigma Aldrich, St. Louis, USA) and subsequently used to normalize the luciferase activity. Transfection normalization was done by beta galactosidase assay. Measurements of mean ± S.D were taken in triplicates and represented graphically on a MS-Excel sheet.

### ELISA

The cell lysates were analyzed for gastrin (gastrin-17 polypeptide) protein by enzyme linked immunoabsorbent assay (ELISA). For ELISA the Gastrin Immuno assay kit (Assay Design Inc, Ann Arbor, USA) were used following the manufacturer's instructions. The total protein concentration in each sample was estimated by Bradford assay (Sigma Aldrich, St. Louis, USA) and was subsequently used to normalize the gastrin value obtained by ELISA.

## Results

### Minimal gastrin promoter contains Smad 3 binding elements

**Figure 1:**
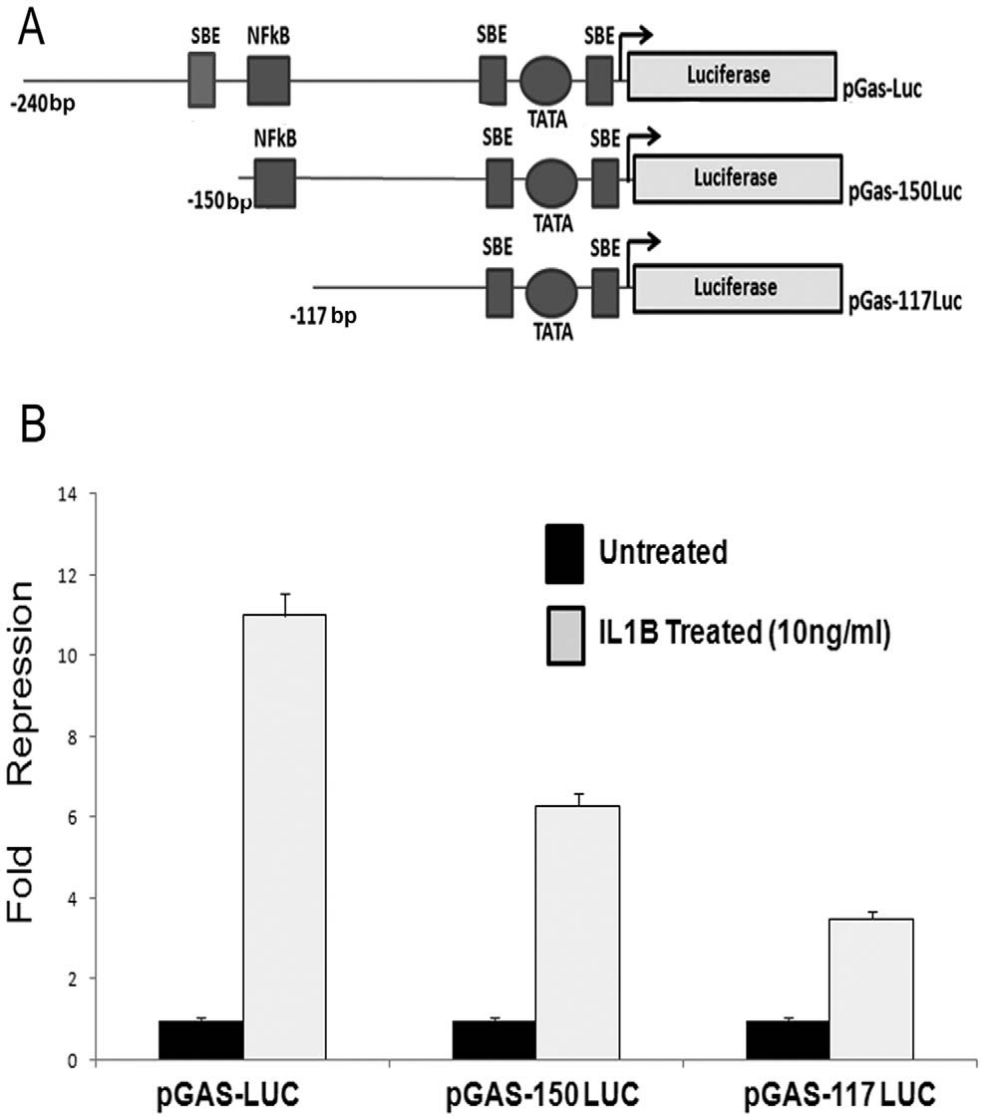
Deletion mapping of 240bp human gastrin promoter for IL1B responsive elements. (A) Schematic diagram of serial deletion of putative gastrin promoter cloned upstream of luciferase reporter gene. The boxes and circle represent different transcription factor binding sites within the 240 bp gastrin promoter. The line diagrams depict the different deletion clones used for the study. (B) The fold inhibition in luciferase activity of different deletion constructs of the gastrin promoter in AGS cells in presence and absence of recombinant IL1B (10 ng/ml). AGS cells were transfected with respective gastrin promoter deletion constructs. Forty-six hours after transfection, cells were either treated or left untreated with 10 ng/ml of IL1B for two hours and then harvested. Luciferase activity was measured and the average fold differences between the treated and untreated sets were plotted from three different experiments.

To identify additional signaling pathways we analyzed the luciferase activity of a series of 5′- deletion mutants of gastrin promoter in presence and absence of recombinant IL1B (10 ng/ml) in AGS cells (Fig. 1A). It was observed that there was steady release of IL1B mediated repression upon serial deletion of the gastrin promoter. On deletion of 90 bp (pGas-150 Luc) from the full length 240 bp (pGas-Luc) gastrin construct, IL1B mediated gastrin repression reduced from 11 fold in pGas-Luc to 6.3 fold in pGas-150 Luc. Moreover, despite the deletion of NFkB binding site (pGas-117 Luc) present within −150 nt to −117 nt there was a 3.6 -fold repression of luciferase activity in presence of IL1B, indicating the presence of other IL1B responsive elements in these regions (Figure 1B). A promoter scan (http://www.gene-regulation.com/pub/programs.html) revealed presence of three putative Smad 3 binding sites in the 240 bp promoter region of human gastrin which might respond to IL1B signaling (Fig. 1A). A previous report stated that a Smad binding element is present in the murine gastrin promoter near its transcription start site [15]. Moreover, gastrin promoter sequence alignment revealed that there is 87% homology in a region just upstream of the start site between murine and human sequences (data not shown). It has been reported that murine gastrin promoter is activated by Smad 3/4 transcription factor [15]. There are also reports that IL1B is able to inhibit Smad 3 responsive genes by inducing Smad 7 ([16], [17], [18]. Therefore, we next looked into the role of Smad 7 in IL1B mediated gastrin repression.

### IL1B inhibits nuclear translocation of Smad 3/Smad 4 complex by inducing Smad 7 in AGS cells

**Figure 2:**
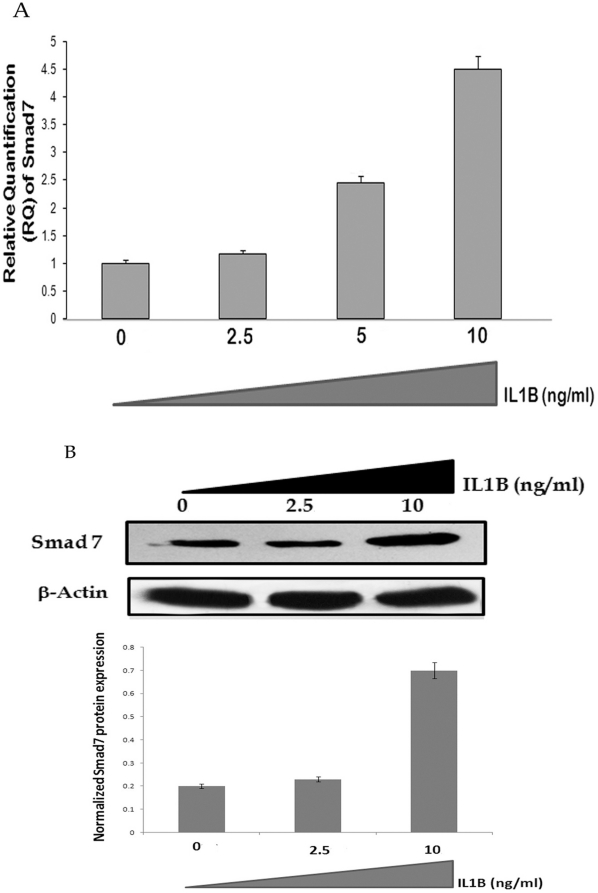
Dose dependent effect of IL1B upon Smad7 expression. (A) Quantitative analysis of IL1B induced *Smad7* mRNA expression. RNA was extracted from AGS cells treated with variable concentration of recombinant IL1B (0, 2.5, 5, 10 ng/ml) for two hours. Real time PCR analysis for *Smad* 7 was performed from cDNA prepared from those samples. Beta-actin was taken as the endogenous control. The graph represents the mean of relative quantification measured from three different experiments +___ SD. (B) Analysis of IL1B induced Smad 7 protein expression. AGS cells were treated with increasing concentration of recombinant IL1B protein for two hours and then lysed and immunoblotted with Smad7 antibody. The bands were scanned by Image J software and normalized band intensity of Smad7 from three different experiments +___ SD was plotted as a histogram. A representative blot is shown.

**Figure 3:**
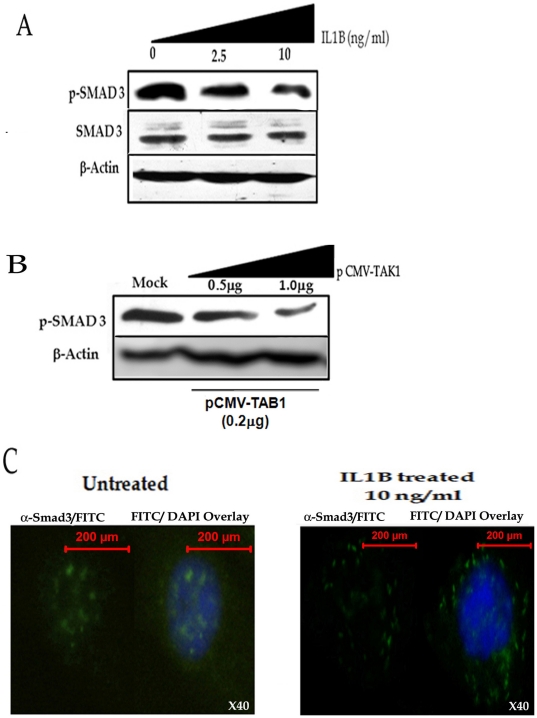
IL1B inhibits Smad 3 phosphorylation thus perturbing its nuclear localization. AGS cells were treated with increasing concentration of recombinant IL1B protein for two hours and then lysed and immuno-blotted with (A) phosphorylated Smad3 specific antibody (p-SMAD 3) and antibody against Smad 3 protein (SMAD 3). A representative blot is shown. (B) AGS cells were co-transfected with different concentrations pCMV-TAK1 and equal amount of pCMV-TAB1, the two immediate downstream mediator of IL1B, and harvested for immuno-blot analysis with p-SMAD3 antibody after forty eight hours. A representative blot is shown. (C) Nuclear localization of Smad 3 in AGS cells treated with or without IL1B (10 ng/ml). In each panel the left section represents FITC conjugated anti-SMAD 3 antibody and the right panel represents the overlay of DAPI/FITC images captured at that field. The experiment establishes that IL1B inhibits nuclear translocation of Smad 3.

We investigated the effects of IL1B on Smad 7 protein and mRNA expression in AGS cells. IL1B was found to significantly up-regulate both Smad 7 mRNA and protein in a dose dependent manner (Fig. 2A and B). It is known that the activated Smad 7 represses Smad 3 responsive gene by inhibiting phosphorylation of the Smad 3 thus interfering with formation of activated Smad 3/4 complex [19]. In the present study we also observed reduced phosphorylation of Smad 3 upon treatment of AGS cells with IL1B (Fig. 3A). However, the total cellular Smad 3 levels remained unaltered (Fig. 3A). One of the key downstream effectors of IL1B signaling pathway is TAK1. Thus, we ectopically expressed pCMVF TAK1 along with its activator TAB1 to show that it also inhibits phosphorylation of Smad 3 in AGS cells (Fig. 3B). Finally, we showed reduced nuclear localization of Smad 3/Smad 4 complex upon treatment of AGS cells with IL1B due to less phosphorylation of Smad 3 (Fig. 3C). Similar results were obtained in AGS cells co-transfected with pCMVF TAK1 along with its activator TAB1 (data not shown).

### IL1B activated Smad 7 represses gastrin expression

**Figure 4:**
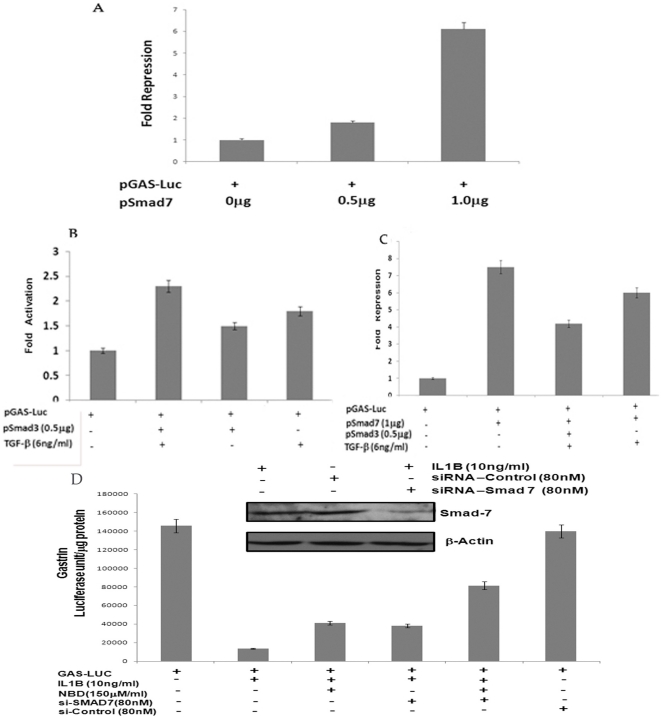
Effect of Smad 7 on gastrin promoter activity. AGS cells were cotransfected with (A) Gas-Luc construct and increasing amount of Smad 7 expression vector (B) Gas-Luc construct and Smad 3 expression vector along with treatment with recombinant TGF-beta for 20 hours (C) Gas-Luc construct and either Smad 7 or both Smad 3 and Smad 7 expression vectors with or without stimulation with recombinant TGF-beta for 20 hours. Cells were harvested 48 hours after transfection and luciferase activity was measured. The mean luciferase activity of three different experiments is represented as fold activation or repression with respect to gastrin promoter alone and graphically plotted. These experiments establish that Smad 7 downregulates gastrin (D) AGS cells were transfected with Gas-Luc construct after the treatment with either si-RNA against Smad 7 or Control scrambled si-RNA for 24 hours. After 44 hours of Gas-Luc transfection, cells were either incubated with NBD (150 µM/ml) or left untreated. Next, cells were stimulated with recombinant IL1B (10 ng/ml) at 46 hours from Gas-Luc transfection or left untreated. Cells were then harvested 48 hours after transfection of Gas-Luc and luciferase activity was measured. The mean luciferase activity of three different experiments is represented as fold activation or repression with respect to gastrin promoter alone and graphically plotted. This experimental observation concludes that both Smad 7 and NFkB pathways contribute equally in IL1B mediated inhibition of gastrin expression. (Inset shows representative Western Blot for Smad7 in Smad 7-siRNA treated and untreated lysates.)

We employed two separate approaches to confirm whether IL1B activated Smad 7 had indeed any effect on gastrin expression (Fig. 4). We co-transfected pGas-Luc with different doses of Smad 7 expression vector in AGS cells and observed that there was a dose dependent decrease in gastrin promoter activity with increasing concentration of Smad 7 expression vector (Fig. 4A). To further validate this finding, we first checked whether gastrin promoter responds to the TGF beta signaling in AGS cells. As shown in Fig. 4B gastrin promoter driven luciferase activity was increased when Smad 3 was ectopically expressed in presence of TGF beta in AGS cells. Next, we co-transfected Smad 3 along with Smad 7 and gastrin luciferase (pGas-Luc) and stimulated AGS cells with TGF beta (6 ng/ml) for 20 hours. We found that stimulation of AGS cells with TGF beta along with overexpression of Smad 3 could alleviate the negative effect of Smad 7 on gastrin luciferase to a significant extent (Fig. 4C). This observation confirms that gastrin luciferase is negatively regulated by Smad 7. Moreover, we can also conclude that the regulation of gastrin depends on the relative molecular abundance of Smad 3 and Smad 7. Previously Chakravorty et al (2009) had reported that Il1B mediated gastrin repression involves NFkB [12]. To investigate the relative contribution of NFkB and Smad7 in IL1B mediated negative regulation of gastrin; we inhibited NFkB activity by its pharmacological inhibitor Nemo Binding Peptide (NBD) or knocked down Smad7 expression by using siRNA against Smad7 and observed its effect on the gastrin promoter. We found that either NFkB or Smad7 inhibitors alone are able to release IL1B mediated gastrin repression by almost about 35% (Fig. 4D). Further, simultaneous inhibition of both these pathways lead to release of the repression by almost about 60% (Fig. 4D). This observation led us to conclude that both these pathways are contributing equally to bring about gastrin repression (Fig. 4D).

### IL1B promoter variant induced Smad 7 differentially downregulates gastrin expression

**Figure 5:**
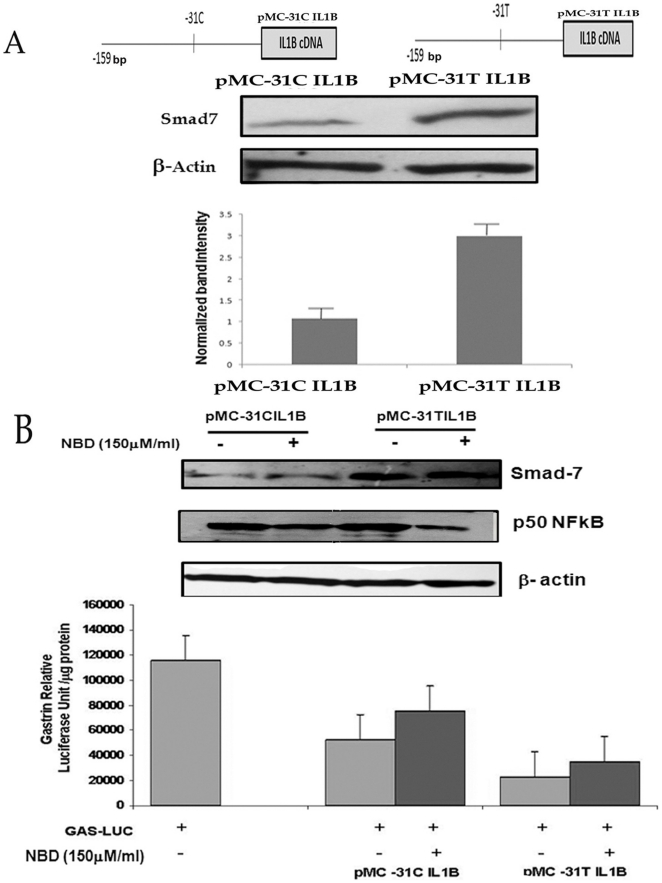
Effect of IL1B promoter polymorphism on Smad 7 expression. (A) *IL1B* promoter polymorphisms differentially regulate Smad 7 protein expression in AGS cells. AGS cells were transfected with the IL1B promoter-driven luciferase construct having either the C or T allele at the −31 position (the constructs are represented as the line diagram) and cells were harvested after 48 hours for immuno blot with Smad7 antibody. The band intensity was scanned and normalized with Beta- actin. The normalized band intensity of three different experiments was plotted. (B) *IL1B* promoter polymorphisms differentially downregulate gastrin via Smad 7, independent of NFkB pathaway. AGS cells were co-transfected with the *IL1B* promoter-driven luciferase construct having either the C or T allele at the −31 position along with pGas-Luc and after 24 hours of transfection cells were either treated with NBD or left untreated. Beta-Gal plasmid was used as transfection control. Cells were harvested after 48 hours of transfection for luciferase assay. Protein in each case was normalized by Bradford assay. The normalized mean RLU/µg protein +_ SD of three different experiments was plotted. The same lysates were also subjected to immunoblot analysis with Smad 7 and NFkB antibody. Beta-actin was used as input control.

Previously we reported that IL1B downregulates gastrin in a promoter variant specific manner and that this differential downregulation is partly due to differential induction of the intermediary signaling molecule NFkB [12]. To investigate similar differential effect of *IL1B* promoter variants on Smad 7 expression, we co-transfected beta galactosidase plasmid along with equal amount of *IL1B -31C* or *IL1B -31T* promoter driven IL1B expression vectors (pMC-IL1B-31C and pMC-IL1B-31T) in AGS cells and Western blot analysis was done with anti-Smad 7 antibody. We observed a 3.5 fold increased activation of Smad 7 expression when IL1B was expressed from IL1B -31T than IL1B-31C expression vector (Fig. 5A). We next established that the differentially expressed IL1B from variant *IL1B* promoters could downregulate gastrin in a predictable pattern through differential activation of Smad 7 in NFkB independent manner (Fig. 5B). We co-transfected AGS cells with pGas-Luc and either pMC-31CIL1B or pMC-31TIL1B and incubated the cells for 24 hours in presence or absence of NFkB inhibitor NBD. We observed that as expected there was *IL1B* promoter variant specific differential downregulation of gastrin (Fig. 5B lower panel), and the intermediary Smad 7 (Fig. 5B upper panel) even when NFkB was inhibited (Fig. 5B upper panel).

**Figure 6:**
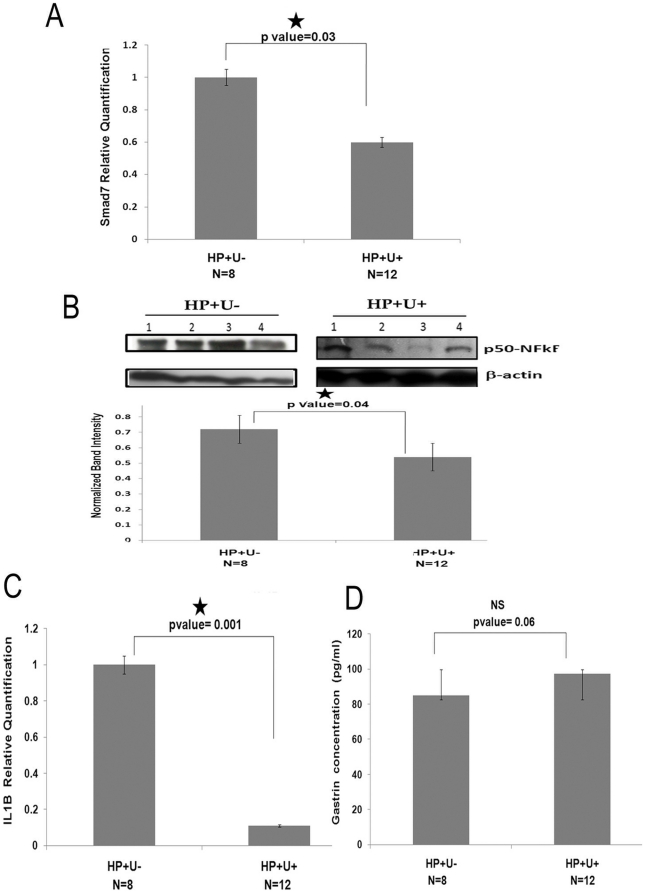
Expression profile of Smad 7, NFkB, IL1B and gastrin in *H. pylori* infected duodenal ulcer patients (HP+U+) and asymptomatic individuals (HP+U−). (A) Smad 7 expression is significantly lower in *H. pylori* infected duodenal ulcer patients (HP+U+) compared to infected asymptomatic individuals (HP+U−): RNA was extracted from gut biopsy samples of *H. pylori* infected duodenal ulcer patients (HP+U+ = 12) and *H. pylori* infected asymptomatic individuals (HP+U− = 8). Real time PCR analysis for Smad 7 mRNA was performed from cDNA prepared from those samples. Beta-actin was taken as endogenous control. The mean relative quantification value from each of the group is represented in the graph. (B) NFkB expression is significantly lower HP+U+ group compared to HP+U-: Gut biopsy samples from both the groups were homogenized and lysed for immuno blot with NFKB p50 antibody. Beta-actin was used as input control. Bradford assay was used to quantify the protein and 50 µg was loaded in each well. The mean normalized value from each of the group is represented in the graph. (C) IL1B expression is significantly lower in HP+U+ group compared to HP+U−: RNA was extracted from gut biopsy samples of both the groups. Real time PCR analysis for IL1B was performed from cDNA prepared from those samples. Beta-actin was taken as the endogenous control. The mean relative quantification value from each of the group is represented in the graph. (D) Gastrin expression is moderately higher in HP+U+ group compared to HP+U−: Samples were homogenized and lysed to measure Gastrin-17 levels by ELISA. Bradford assay was used to quantify the protein. The normalized mean value obtained from each group is graphically plotted.

Next, we sought to understand the clinical significance of differential Smad 7 expression in *H. pylori* associated duodenal ulcer patients. We compared the expression of Smad 7, NFkB, IL1B and gastrin in antral gut biopsy samples between H. pylori infected asymptomatic and symptomatic individuals (HP+U− vs. HP+U+). Quantitative RT-PCR analysis of *Smad 7* mRNA showed significantly lower expression of *Smad 7* in ulcer patients compared to asymptomatic individuals despite both being infected with *H. pylori* (Fig. 6A). Similar result was also obtained in case of NFkB levels as measured by Western blot analysis of antral gut biopsy samples from these two groups of individuals (Fig. 6B). Interestingly, these results corroborated with the expression of *IL1B* in the gut biopsy samples of these two groups. *H. pylori* associated ulcer patients have significantly lower level of *IL1B* than infected asymptomatic individuals (Fig. 6C). Finally, measurement of antral gastrin levels by ELISA in these two sets of samples revealed a higher level in HP+U+ samples than that of HP+U− though it is not statistically significant (Fig. 6D).

## Discussion

**Figure 7:**
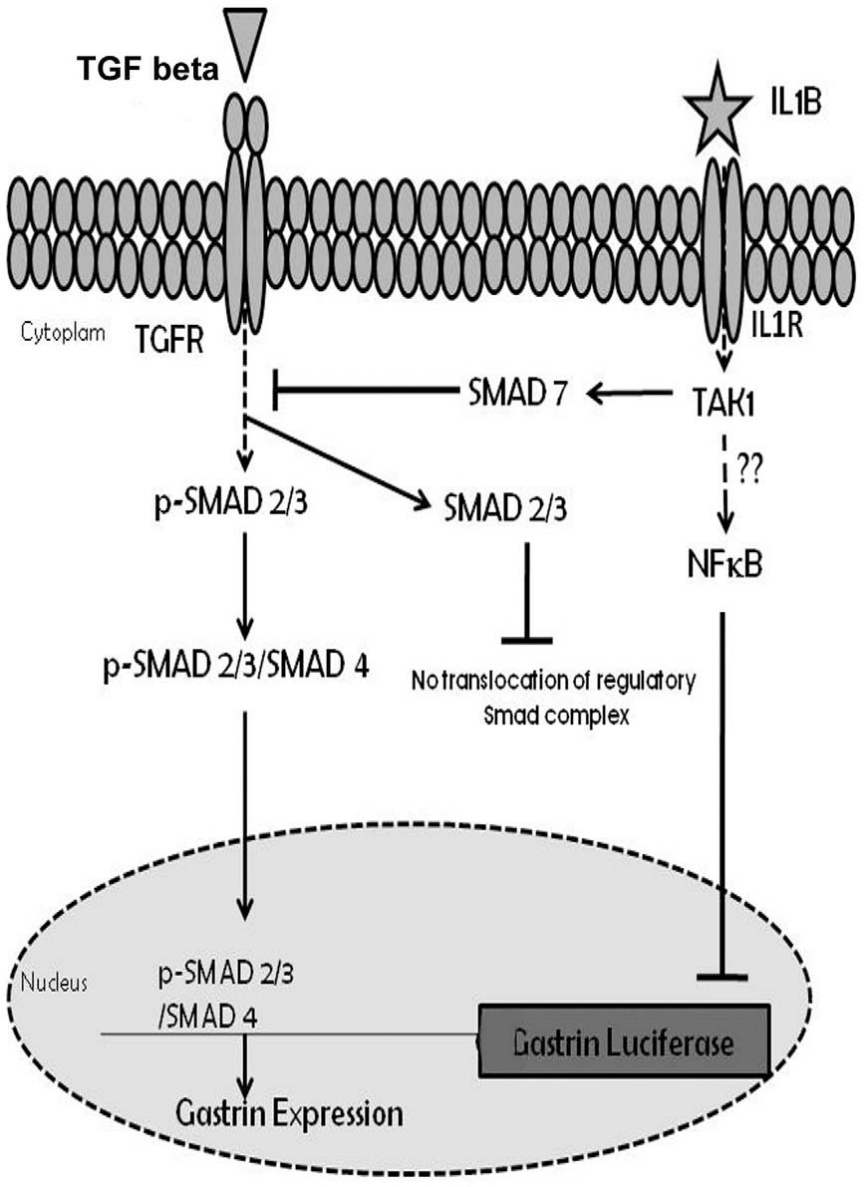
IL1B activates two different pathways having a cumulative effect on Gastrin repression. Stimulation with IL1B results in an increase in Smad7 levels which inhibits the TGF-beta pathway by blocking the nuclear localization of the regulatory Smad 2/3/4 complex. On the other hand, IL1B also activates the NFKB pathway which plays a direct role in repression of the gastrin gene expression. Dotted arrows represent pathways that are yet to be established.

It has been reported that *H. pylori* elicited IL1B is one of the various modulators of the gastrin–somatostain hormone axis which regulates the acid secretion in gut [7]. In this study, we illustrate one of the probable pathways by which the proinflammatory cytokine IL1B affects gastrin expression. We show that IL1B induced Smad 7 inhibits the nuclear localization of Smad 3, thereby bringing about transcriptional repression of Smad 3 dependent gastrin expression. The -31C promoter polymorphism of *IL1B* has been reported to be associated with *H. pylori* mediated duodenal ulcer in various populations [10]. We also observed that there was promoter variant specific differential induction of Smad 7 expression and gastrin repression by IL1B. We have further measured the expression of Smad 7, NFkB, IL1B and gastrin in *H. pylori* infected duodenal ulcer patients and asymptomatic individuals and validated our hypothesis that differential expression of IL1B may lead to differential level of Smad 7 and NFkB causing altered gastrin level *in vivo* (Fig. 7).

It is known that IL1B acts as a potent inhibitor of gastric acid secretion, on a molar basis, and is estimated to be 100-fold more potent than proton pump inhibitors and 6,000-fold more potent than H_2_ antagonists [9]. Kondo et al reported that IL1B is an extremely effective acid suppressant when given intracisternally and it also inhibits release of histamine from gastric enterochromaffin-like cells [20]. Despite many *in vivo* studies showing inhibition of acid secretion by the cytokines, the mechanism of the inhibition by IL1B is poorly understood. We had previously reported that IL1B inhibits acid secreting hormone gastrin by activating NFkB [12]. However, the contribution of NFkB activation to gastrin downregulation was partial [12]. We identified Smad 3 binding elements within the 240 bp promoter region of human gastrin in the present study. It is known that regulation of Smad 3 is mediated by TGF- beta signaling [21]. TGF beta induced activated TGF-betaRI phosphorylates Smad2 and Smad 3. Phosphorylated Smad 2 and Smad 3 associate with Smad 4 and translocate to the nucleus where Smad proteins participate in transcriptional control of target gene [21]. Thus, disruption of TGF beta signaling would negatively influence Smad 3 target gene gastrin expression [22]. It has been reported that, TGF beta mediated signalling pathway is impaired in cells with high expression of Smad 7 because it prevents Smad 2/3 phosphorylation following the binding of active TGF beta 1 to the receptor [23]. There are several reports which suggests that pro-inflammatory cytokines like TNF-α, IL1B and IFNγ which are produced in excess in mucosa during *H. pylori* infection, can positively influence Smad 7 expression [16], [18], [24], [25], [26]. Similarly, we also observed that in gastric cancer cell line AGS, IL1B induced Smad 7, inhibits the phosphorylation of Smad 3 and subsequently interferes with gastrin expression. A previous report also showed that failure in TGF beta signaling plays an important role in gut inflammation and is associated with high expression of Smad 7 [27], [28]. Though there are no reports on association of TGF beta with duodenal ulcer, a positive correlation between TGF beta expression and gastric ulcer healing has been reported. Patients with healed gastric ulcers showed an increased expression of both TGF beta and its receptors while patients with refractory ulcers had weak or deficient TGF beta expression in the gastric mucosa, suggesting crucial role of TGF beta in gastric ulcer healing [29].

Our clinical data suggest that individuals with *H.pylori* associated duodenal ulcer have higher expression of gastrin and a correlated lower expression of NFkB and Smad 7. Higher expression of gastrin would also lead to higher acid output. The difference in IL1B expression in *H.pylori* infected asymptomatic individuals (Hp+ U−) in comparison to duodenal ulcer patients (HP+U+) can be attributed to the difference in genetic make up of these individuals as reported by various association studies. These studies along with previous data from our lab have reported that IL1B -31C promoter variant is associated with *H. pylori* infected duodenal ulcer. In our previous report, we have also observed that individuals homozygous for -31C IL1B variant, irrespective of *H. pylori* status have lower expression of IL1B than individuals harboring the alternative genotype [10], [12]. However in this study, we observe that the difference in gastrin expression between infected symptomatic (HP+U+) and asymptomatic (HP+U−) is not statistically significant. This apparent anomaly could be due to small sample size and/or existence of other IL1B independent pathway controlling gastrin expression and acid secretion. Thus, the *in vivo* scenario with respect to relative levels of Smad 7, NFkB, IL1B and gastrin in gut biopsy samples of *H. pylori* infected asymptomatic and duodenal ulcer patients reflects the *in vitro* observation that differential expression of IL1B can modulate differentially the expression of both Smad 7 (this study) and NFkB (this study and [12]) which in turn may alter the gastrin levels in human gut in a predictable manner. This is significant because it emphasizes that the subtle difference in signaling due to promoter polymorphisms can alter the course of biochemical pathway resulting in variation in disease phenotype.
